# Food allergy in the Netherlands: differences in clinical severity, causative foods, sensitization and DBPCFC between community and outpatients

**DOI:** 10.1186/s13601-015-0051-1

**Published:** 2015-02-25

**Authors:** Thuy-My Le, Els van Hoffen, Ischa Kummeling, James Potts, Barbara K Ballmer-Weber, Carla AFM Bruijnzeel-Koomen, Ans FM Lebens, Jonas Lidholm, Titia M Lindner, Alan Mackie, EN Clare Mills, Ronald van Ree, Stefan Vieths, Montserrat Fernández-Rivas, Peter G Burney, André C Knulst

**Affiliations:** Department of Dermatology/Allergology, University Medical Center Utrecht, PO Box 85500, 3508 GA Utrecht, The Netherlands; Department of Respiratory Epidemiology, Occupational Medicine and Public Health, National Heart and Lung Institute, Imperial College London, London, UK; Allergy Unit, Department of Dermatology, University Hospital Zürich, Zürich, Switzerland; Thermo Fisher Scientific, Uppsala, Sweden; Institute of Food Research, Norwich Research Park, Colney, Norwich, NR4 7UA UK; Institute of Inflammation and Repair, Manchester Academic Health Sciences Centre, Manchester Institute of Biotechnology, The University of Manchester, Manchester, UK; Department of Experimental Immunology and Department of Otorhinolaryngology, Academic Medical Center of the University of Amsterdam, Amsterdam, The Netherlands; Division of Allergology, Paul-Ehrlich-Institut, Federal Institute for Vaccines and Biomedicines, Langen, Germany; Allergy Department, Hospital Clinico San Carlos, IdISSC, Madrid, Spain; Current affiliation: NIZO food research, Ede, The Netherlands

**Keywords:** Adults, Community, DBPCFC, Food allergy, sIgE

## Abstract

**Background:**

It is unknown whether food allergy (FA) in an unselected population is comparable to those from an outpatient clinic population.

**Objective:**

To discover if FA in a random sample from the Dutch community is comparable to that of outpatients.

**Methods:**

This study was part of the Europrevall-project. A random sample of 6600 adults received a questionnaire. Those with symptoms to one of 24 defined priority foods were tested for sIgE. Participants with a positive case history and elevated sIgE were evaluated by double-blind placebo-controlled food challenge (DBPCFC). Outpatients with a suspicion of FA were evaluated by questionnaire, sIgE and DBPCFC.

**Results:**

In the community, severe symptoms were reported less often than in outpatients (39.3% *vs.* 54.3%). Participants in the community were less commonly sensitized to any of the foods. When selecting only those with a probable FA (i.e. symptoms of priority food and elevation of sIgE to the respective food), no major differences were observed with respect to severity, causative foods, sensitization and DBPCFC between the groups.

**Conclusion:**

In the Netherlands, there are large differences in self-reported FA between community and outpatients. However, Dutch community and outpatients with a probable FA do not differ with respect to severity, causative foods, sensitization and DBPCFC-outcome.

## Background

A recent meta-analysis showed that self-reported prevalence of food allergy (FA) ranged from 3% to 35% in adults [1]. However, the prevalence of FA as diagnosed by double-blind placebo-controlled food challenge (DBPCFC) is estimated to be around 2% to 4% for adults [2-7]. A study from a regional allergy center in the UK, serving a population of 1.6 million, reported a population prevalence of 0.03%, being a factor of 100 less than in population-based studies. This suggests that a minority of FA patients present to their GP and are subsequently referred [8].

Most studies investigating the clinical aspects of FA involve patients from a (tertiary) allergy clinic. Since only a fraction of food allergic individuals from the general population visit a doctor, [8] it is not clear how representative results from such studies are for the general population. However, studies comparing FA between the community and outpatients are lacking.

The aim of this study was to investigate to what extent FA in a random sample of the Dutch community is comparable to FA in outpatients.

## Methods

This study was part of the EuroPrevall-project, a multidisciplinary European project investigating several aspects of FA [9]. It consisted, among many different, of an epidemiological element investigating the prevalence of FA in the community and an outpatient clinical element. This study was approved by the local medical ethical committee (METC) of the University Medical Center Utrecht.

### Community

The epidemiological study consisted of three stages and is described in detail elsewhere [10]. In short, the study took place from October 2006 until March 2009. The 1st stage was a population-based study in which a short questionnaire to screen for FA was sent out to a random sample of 6600 adults aged 20–54 years living in the city of Utrecht, The Netherlands.

In the 2nd stage, all those who in the screening questionnaire reported to have experienced adverse reactions to one of the 24 preselected priority foods (hen’s egg, cow’s milk, peanut, soy, hazelnut, walnut, celery, kiwi, apple, peach, sesame, mustard, wheat, fish and shrimp, buckwheat, corn, carrot, tomato, melon, banana, lentils, sunflower, poppy seeds) were invited to come to the hospital for a detailed questionnaire and serum IgE testing .

In the 3rd stage, participants with self-reported symptoms and sIgE to at least one of the priority foods were called in for DBPCFC and a full clinical evaluation, identical to that of the outpatients (see below).

### Outpatients

The outpatient study took place from January 2006 until June 2009. All adult patients (age 18 years and older) who were referred to our tertiary allergy centre with a suspected FA, were asked to participate in the EuroPrevall outpatient clinic study. Inclusion criteria were symptoms developing within 2 hours after ingestion of a food. Participating outpatients underwent a clinical evaluation comprising a thorough medical history using a standardized questionnaire, sIgE testing and DBPCFC.

### Clinical evaluation

All clinical information was collected following a standardized questionnaire which was specifically developed for the Europrevall-study. Symptoms were classified into mild (symptoms of the oral cavity), moderate (gastro-intestinal symptoms and symptoms of the skin and mucous membranes) and severe (respiratory or cardiovascular symptoms).

A probable FA was defined as symptoms within 2 hours and sIgE ≥0.35 kU_A_/L to a priority food. Although DBPCFC is the gold standard for diagnosis of FA, diagnosis of FA in majority of patients in clinical practice is based on medical history and sIgE. Therefore, in this study we chose a pragmatical approach using a suggestive history (i.e. symptoms <2 hours of ingestion) together with IgE sensitization to define a probable FA.

### In vitro diagnosis

sIgE testing for all priority foods were performed in a single laboratory using the ImmunoCAP system and reagents from Phadia (Thermo Fisher Scientific, Uppsala, Sweden). sIgE values of ≥0.35 kU_A_/L were regarded as positive.

### DBPCFC

All participants with symptoms to hen’s egg, cow’s milk, peanut, hazelnut, celery, apple, peach, fish or shrimp were asked to participate in DBPCFC. A detailed protocol of the EuroPrevall DBFCFC is described elsewhere [11]. Briefly, active and placebo provocations were randomly performed on two different days with eight increasing doses. The single doses were administered at an interval of at least 20 minutes up to the top dose. The interval could be extended at the request of the patient, according to the case history of the patient, or in case of severe persisting subjective symptoms. Challenges were discontinued after the dose leading to the first objective allergic symptoms or ingestion of the whole meal. Objective symptoms considered for discontinuation of the provocation were blisters of the oral mucosa, skin symptoms such as flush, urticaria, angioedema, rhinitis, conjunctivitis, drop of blood pressure of at least 20 mmHg, drop of FEV1 > 12% or PEF ≥ 20%, laryngeal oedema, diarrhoea, emesis or in case of severe persistent subjective symptoms lasting for more than 45 minutes such as severe itching of palms, soles, head or severe gastric/abdominal pain. If all DBPCFC doses were negative, patients underwent an open food challenge. Patients that had a reaction of any type and any duration on the active day and no reaction on the placebo were classified as reactors, patients that did not react on the active nor the placebo day were tolerant and patients that reacted on the placebo day were placebo responders.

### Statistics

Chi-square tests and, where appropriate, Fisher’s exact tests were used to test the differences in several clinical characteristics (i.e. gender, severity of symptoms, causative foods, atopic diseases, sensitization and DBPCFC outcome) between the community and outpatients. Differences in age and the number of causative foods between the two groups were calculated using Student’s T-test and, where appropriate, Mann–Whitney U test. Statistical analysis was performed using SPSS version 20 (SPSS Inc., Chicago, Illinois, USA) for Windows.

## Results

### Study population

**Figure 1:**
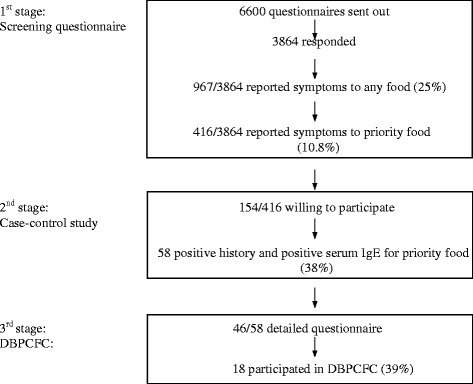
**Flow-diagram of the epidemiological part of the study in the community.**

Of the screening questionnaires that were sent out to a random sample of 6600 adults, 3864 responded, of whom 967 (25.0%) reported adverse food reactions to any food and 416 (10.8%) to at least one priority food (Figure 1). 154 of these 416 (37.0%) were willing to come to our clinic for the second stage consisting of a detailed questionnaire and sIgE testing. In 37.7% (58/154) sIgE was positive for the respective food. Of these 58 cases, 46 entered the 3rd stage for a full clinical evaluation.

In the outpatient clinical part of the study, all outpatients that met the inclusion criteria agreed to participate in the study. In total, 133 outpatients that were referred to our allergy center were included during the study period. Symptoms to at least one priority food were reported by 127 outpatients, in 102 of which sIgE to the same food(s) was detected.

### Reported adverse food reactions to any food in the community

Adverse reactions to any food were reported by 25.0% of the responders in the community. Cow’s milk (10.7%), nuts (9.5%), apple (9.4%) and fish (6.3%) were the most commonly reported causative foods. Mild symptoms (consisting of only oral allergy symptoms) were reported by 10.1%, moderate symptoms by 58.4% and severe symptoms by 21.4% of participants. In the group with moderate symptoms, gastro-intestinal symptoms dominated (60.2%).

Of the 967 participants reporting adverse reactions to any food, 416 reported symptoms to priority food and 551 reported symptoms to other foods. These two groups were compared and showed that the group with symptoms to priority food significantly more often reported oral allergy symptoms (51.5% vs. 24%), difficulty with swallowing (15.9% vs. 6.3%) and breathlessness (11.3% vs. 6.8%). In the group with symptoms other than priority food gastro-intestinal symptoms dominated and was significantly higher than in the priority food group (47.7% vs. 38.6%). Furthermore, the group without symptoms of priority food significantly more often reported that the symptoms to the culprit food took place once only (26.8%).

### Reported adverse reactions to priority foods in community and outpatients

**Figure 2:**
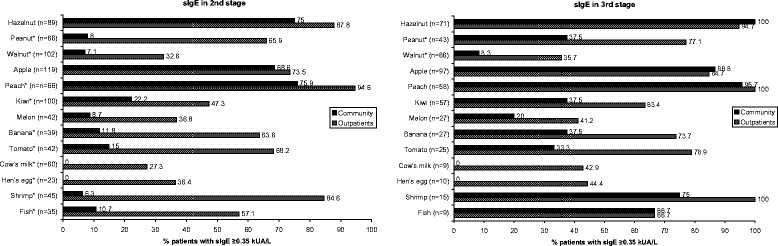
**Percentage of positive sIgE in the community and outpatients with symptoms to the food in the 2nd stage (reported symptoms) and 3rd stage (reported symptoms and positive sIgE for at least 1 food) of the study.** The number (n = ..) between brackets indicates the total number of the community and outpatients that reported symptoms to that respective priority food. *p-value <0.05.

Of the participants reporting adverse reactions to any food (n = 967), 43.0% reported symptoms to at least one of the priority foods. Walnut, apple, cow’s milk, hazelnut and kiwi were the most commonly reported priority foods in the community (Table 1, 2nd stage). The frequency of severe symptoms rose from 21.4% in the 1st stage to 39.3% in the 2nd stage in participants from the community. Remarkably, only a small percentage of participants from the community who reported symptoms to priority foods also had a positive sIgE for the respective food (Figure 2).

**Table 1:** **Patient characteristics of the three different stages**

	**1st stage (possible FA to any food)** ^**||**^	**2nd stage (possible FA to priority food)** ^**‡**^			**3rd stage: (probable FA to priority food)** ^**§**^		
	**Community (n = 967) %**	**Community (n = 154) %**	**Outpatients (n = 127) %**	**p-value**	**Community (n = 46) %**	**Outpatients (n = 102) %**	**p-value**
Male gender	32.0	31.2	35.4	0.52	30.4	38.2	0.36
Age (mean ± SD)	34.7 (±9.3)	34.1 (±9.2)	32.2 (±12.3)	0.16	35.3	30.4	0.01*
Number of foods causing symptoms (median (range))		4 (1-17)	5 (1-18)	0.40	6 (1-14)	6 (1-18)	0.96
Symptom^¦^:							
-mild	10.1	14.0	26.0	0.01*	26.1	21.0	0.50
-moderate	58.4	46.7	19.7	<0.001*	28.3	19.0	0.21
-severe	21.4	39.3	54.3	0.008*	45.7	60.0	0.11
Atopy:							
-pollen allergy		70.8	94.5	<0.001*	97.8	94.1	0.44
-dermatitis		36.4	33.3	0.61	25.6	37.8	0.18
Symptoms of plant food							
-Hazelnut	2.4	31.2	32.3	0.84	71.7	37.3	<0.001*
-Peanut	2.7	16.2	32.3	0.002*	18.6	34.3	0.04*
-Walnut	2.5	36.4	36.2	0.98	52.2	41.2	0.21
-Apple	9.4	33.1	53.5	0.001*	82.6	57.8	0.003*
-Peach	1.0	18.8	29.1	0.04*	50.0	34.3	0.07
-Kiwi	4.8	29.2	43.3	0.01*	34.8	40.2	0.53
-Melon	1.2	14.9	15.0	1.00	21.7	16.7	0.46
-Banana	1.4	11.0	17.3	0.17	17.4	18.6	0.86
-Tomato	2.8	13.0	17.3	0.31	13.0	18.6	0.40
-Carrot	1.8	8.4	11.8	0.42	19.6	14.7	0.46
Symptoms of animal food							
-Cow’s milk	10.7	31.8	8.7	<0.001*	4.3	6.9	0.72
-Hen’s egg	2.4	7.8	8.7	0.79	2.2	8.8	0.17
-Fish	6.3	18.2	5.5	0.001*	6.5	5.9	1.00
-Shrimp	3.7	20.8	10.2	0.02*	8.7	10.8	0.78

FA = food allergy.

^¦^mild = oral allergy symptoms, moderate = gastro-intestinal symptoms or symptoms of the skin and mucous membranes, severe = respiratory or cardiovascular symptoms.

^||^reported adverse food reaction to any food.

^‡^positive history for priority food.

^§^positive history and serum IgE for priority food.

*p-value < 0.05.

When comparing the community to outpatients, using reported symptoms to priority food as selection in both groups, it appeared that severe symptoms were significantly more common in outpatients (54.3% *vs.* 39.3%, p = 0.008). The frequency in which the different priority foods were reported, differed significantly for some foods: in the community the frequency of allergy to cow’s milk, fish and shrimp was significantly higher, whereas outpatients reported symptoms to peanut, apple, peach and kiwi at a significantly higher frequency (Table 1, 2nd stage). Outpatients had significantly more clinically relevant sIgE sensitisation compared to the community for all foods, except for hazelnut and apple, where no difference was seen between both groups (Figure 2).

In summary, causative foods, severity and relevant food sensitization differed between the community and outpatients when only selecting on self-reported symptoms to priority foods.

### Probable FA in community and outpatients

Of the participants from the community that reported symptoms to a priority food, 58/154 (37.7%) also had a positive sIgE for that food. These participants were considered to have a probable FA. When comparing the community participants and outpatients, using symptoms and sIgE to priority food as selection in both groups (i.e. probable FA), the differences in causative foods as seen in the 2nd stage disappeared largely. Only hazelnut and apple were significantly more frequently reported as a causative food in the community, whereas peanut was more commonly reported in outpatients (Table 1). In contrast to the 2nd stage, no differences were seen in the severity of symptoms between the community and outpatients (Table 1). Whereas in the 2nd stage major differences were observed between the two groups in sensitization to most priority foods, these differences disappeared largely when selecting on FA (Figure 2). Table 2 shows the clinical characteristics in more detail for the main foods. The majority with a probable food allergy were birch pollen sensitized, had symptoms within a few minutes after ingestion of the culprit food and oral allergy symptoms was the most common symptom. A probable cow’s milk en hen’s egg allergy was rare in our adult population. In those with a cow’s milk and hen’s egg allergy anaphylaxis was relatively frequent (25%).

**Table 2:** **Clinical characteristerics per food in patients with a probable food allergy (i.e. symptoms and specific IgE)**

	**Community**													**Outpatients**												
		**Symptoms**						**Time interval**				**sIgE food**	Birch IgE*		**Symptoms**						**Time interval**				**sIgE food**	Birch IgE*
	**N**	**OAS (%)**	**skin (%)**	**GI (%)**	**Resp (%)**	**Cardio (%)**	**Anaph (%)**	**<5 min**	**5-30 min**	**30-120 min**	**>2 hrs**	**Median (Range)**	**(%)**	**N**	**OAS (%)**	**skin (%)**	**GI (%)**	**Resp (%)**	**Cardio (%)**	**Anaph (%)**	**<5 min**	**5-30 min**	**30-120 min**	**>2 hrs**	**Median (range)**	**(%)**
Hazelnut	33	90.9	27.3	6.1	42.4	0	0	84.8	9.4	6.3	0	12.5 (0.47-74.99)	72.7	36	97.2	27.8	27.8	47.2	2.8	5.6	86.1	13.9	0	0	23.37 (0.65-196.54)	97.2
Peanut	3	66.7	33.3	33.3	33.3	0	0	50	50	0	0	2.63 (0.60-5.58)	100	27	88.9	59.3	29.6	48.1	0	0	77.8	22.2	0	0	2.21 (0.35-321.78)	85.2
Walnut	2	100	50	50	50	0	0	100	0	0	0	1.04 (0.51-1.58)	100	15	93.3	60	20	26.7	6.7	6.7	93.3	0	6.7	0	5.53 (0.36-27.56)	93.3
Apple	33	97	24.2	21.2	24.2	0	0	75.8	21.2	3	0	1.49 (0.37-21.18)	69.7	51	100	28	16	30	0	0	77.6	22.4	0	0	2.03 (0.42-31.19)	100
Peach	22	95.5	31.8	13.6	27.3	0	0	77.3	18.2	4.5	0	2.00 (0.53-12.91)	72.7	36	97.1	31.4	17.1	37.1	0	0	77.1	22.9	0	0	3.79 (0.59-44.56)	100
Kiwi	6	100	33.3	16.7	16.7	0	0	100	0	0	0	1.07 (0.40-11.21)	83.3	26	96.2	19.2	19.2	26.9	0	0	80	20	0	0	1.67 (0.35-152.57)	88.5
Cow’s milk	0	-	-	-	-	-	-	-	-	-	-	-	-	4	50	50	50	25	25	25	50	25	0	0	10.05 (0.43-34.12)	50
Hen’s egg	0	-	-	-	-	-	-	-	-	-	-	-	-	4	75	50	50	25	0	25	25	50	25	0	1.68 (0.40-3.04)	75

OAS = oral allergy symptoms, GI = gastro-intestinal, Resp = respiratory, cardio = cardiovascular, anaph = anaphylaxis.

*Birch sIgE = sIgE birch ≥0.35.

In total 18 participants from the community and 30 outpatients underwent DBPCFC. There were no significant differences in the severity of symptoms for the individual foods between those who agreed to participate in DBPCFC and those who declined (p > 0.05, data not shown). The percentage of positive DPBCFC did not differ between community and outpatients (77.8% *vs.* 63.3%, p = 0.47). Of the patients with a positive DBPCFC, 35.7% of the community and 31.6% of outpatients (p = 0.80) had objective signs. In both groups, the percentage of placebo reactors was high: 16.7% in the community and 20% in the outpatients. All placebo reactors reported subjective symptoms and not objective signs on placebo day. The outcome of the challenges per food are shown in Table 3.

**Table 3:** **Double-blind placebo-controlled food challenge (DBPCFC) in community and outpatients**

	**Community**						**Outpatients**					
	**N**	**Positive DBPCFC (%)**	**Negative DBPCFC (%)**	**Placebo reactor (%)**	Objective signs* (%)	Subjective symptoms* (%)	**N**	**Positive DBPCFC (%)**	**Negative DBPCFC (%)**	**Placebo reactor (%)**	Objective signs* (%)	Subjective symptoms* (%)
Hazelnut	9	77.8	11.1	11.1	42.9	100	10	60	20	20	50	100
Peanut	1	100	0	0	0	100	9	66.7	22.2	11.1	50	100
Apple	5	60	0	40	33.3	66.7	8	50	12.5	37.5	0	100
Peach	2	100	0	0	0	100	1	100	0	0	0	100
Celery	1	100	0	0	100	100	0	-	-	-	-	-
Cow’s milk	0	-	-	-	-	-	1	100	0	0	0	100
Hen’s egg	0	-	-	-	-	-	1	100	0	0	0	100

* This is calculated as a percentage from those with a positive DBPCFC.

In conclusion, when focusing on a probable FA, no difference could be demonstrated between the community participants and the outpatients with regard to severity, causative foods, relevant food sensitization and DBPCFC-outcome.

## Discussion

Since most studies investigating FA use outpatients as a study population, the question arises whether such study results are applicable to food allergic persons in the community.

To our knowledge this is the first study to compare FA in the community and outpatients. In this study we found that, when focusing on self-reported symptoms to priority foods, there are large differences in causative foods, severity and relevant food sensitization between community and outpatients. However, when selecting those with a probable FA, as defined by reported symptoms to priority food together with a positive sIgE to the respective food, it became evident that the differences in the community and outpatients disappeared. This indicates that patients with a probable FA seen in a tertiary outpatient allergy center are not different from those with a probable FA in the community.

Previous population based studies showed that the prevalence of self-reported FA to any food varied from 3% to as high as 35%, whereas the prevalence of true FA is estimated to be 2-4% [1-7,12-14]. In our study we confirmed for the Dutch population the discrepancy between the prevalence of self-reported adverse food reactions (10.8%), a FA as defined by a suggestive history and supported by sIgE (4.1%) and a FA confirmed by DBPCFC (3.2%).

Since non-response bias could play a role in the community survey, we performed a non-response analysis. The response rate in the 1st stage of the community survey was 61%. We calculated the cumulative prevalence of adverse food reaction at the time of response to extrapolate the prevalence to non-responders [15]. This showed that the cumulative prevalence of adverse food reaction stabilized after a response rate of 40%, indicating that non-response bias does not play a major role in the 1st stage of the study. For the 2nd stage, we compared age, gender, doctor-diagnosed FA and the symptoms between responders and non-responders. Only the frequency of oral allergy symptoms differed, being higher in the responders (61% *vs* 49%, p = 0.02*)*, which could indicate that responders from the 2nd stage of the study were more likely to have a probable food allergy compared to non-responders. This would mean that the differences found between community and outpatients in the 2nd stage would even be larger. For the 3rd stage, no differences were seen between responders and non-responders with regard to age, sex and symptoms (data not shown). Although the response rate was low, the profile of responders and non-responders were largely similar, and therefore it is thought that a possible non-response bias would not play a major role. Low response rates are not unusual in population based studies investigating the prevalence of food allergy [3,5].

The gold standard for the diagnosis of FA is DBPCFC [16]. However, DBPCFC is time-consuming, expensive, subjects patients to potential severe allergic reactions, requires the need for well equipped facilities and may not be able to reproduce the conditions that occurred when the reported allergic reaction took place [17,18]. Due to these practical problems, in normal clinical settings FA is often diagnosed by a thorough medical history and a test for sensitization [17,19]. In our study, medical history and sIgE was used to define a probable FA since this could be performed in all participants and outpatients. Certain participants in the community and outpatient studies agreed to undergo DBPCFC and, whilst a relatively small sample, no differences were seen in the rates of either positive DBPCFC (78% and 64%, respectively) or the objective signs between the two groups. These data indicate that the clinical characteristics of individuals with a probable FA in this population were similar between the two populations and that the estimates of prevalence relying only on clinical history and sIgE will over-estimate rates of confirmed food allergy by around 22-37%. A low participation rate in DBPCFC is also reported in other studies [5,7]. The main reason for refusal to participate in DBPCFC in our patients was the lack of time.

In general, it might be expected that the frequency of severe FA in outpatients referred to a tertiary allergy center is higher compared to the community. In this study we observed no significant difference in the frequency of severe symptoms between both groups. Of the participants with a probable FA in the community, 40.4% had never sought medical care for their symptoms to food and of these, 46.4% had severe symptoms upon ingestion of the causative food (data not shown). Thus, severe symptoms are not always a trigger for FA-patients to seek medical care.

## Conclusions

In the Netherlands, there are large differences in self-reported FA between community and outpatients. However, Dutch community and outpatients with a probable FA do not differ with respect to severity, causative foods, sensitization and DBPCFC-outcome.

## Abbreviations

DBPCFC: Double-blind placebo-controlled food challenge
FA: Food allergy
OR: Odds ratio
